# Intrapulmonary percussive ventilation in acute exacerbations of COPD patients with mild respiratory acidosis: a randomized controlled trial [ISRCTN17802078]

**DOI:** 10.1186/cc3724

**Published:** 2005-06-01

**Authors:** Frédéric Vargas, Hoang Nam Bui, Alexandre Boyer, Louis Rachid Salmi, Georges Gbikpi-Benissan, Hervé Guenard, Didier Gruson, Gilles Hilbert

**Affiliations:** 1 Département de Réanimation Médicale, Hôpital Pellegrin-Tripode, Bordeaux, France; 2 Laboratoire de Physiologie EA 518, Université Victor Segalen Bordeaux 2, Bordeaux, France; 3 Institut Fédératif de Recherche de Santé Publique (IFR-99), Université Victor Segalen Bordeaux 2, Bordeaux, France

## Abstract

**Introduction:**

We hypothesized that the use of intrapulmonary percussive ventilation (IPV), a technique designed to improve mucus clearance, could prove effective in avoiding further deterioration in patients with acute exacerbations of chronic obstructive pulmonary disease (COPD) with mild respiratory acidosis.

**Methods:**

The study was performed in a medical intensive care unit of a university hospital. Thirty-three patients with exacerbations of COPD with a respiratory frequency ≥ 25/min, a PaCO_2 _> 45 Torr and 7.35 ≤ pH ≤ 7.38 were included in the study. Patients were randomly assigned to receive either standard treatment (control group) or standard treatment plus IPV (IPV group). The IPV group underwent two daily sessions of 30 minutes performed by a chest physiotherapist through a full face mask. The therapy was considered successful when both worsening of the exacerbation and a decrease in pH to under 7.35, which would have required non-invasive ventilation, were avoided.

**Results:**

Thirty minutes of IPV led to a significant decrease in respiratory rate, an increase in PaO_2 _and a decrease in PaCO_2 _(p < 0.05). Exacerbation worsened in 6 out of 17 patients in the control group versus 0 out of 16 in the IPV group (p < 0.05). The hospital stay was significantly shorter in the IPV group than in the control group (6.8 ± 1.0 vs. 7.9 ± 1.3 days, p < 0.05).

**Conclusion:**

IPV is a safe technique and may prevent further deterioration in patients with acute exacerbations of COPD with mild respiratory acidosis.

## Introduction

Acute exacerbations of chronic obstructive pulmonary disease (COPD) are a frequent cause of admission to hospital and the intensive care unit (ICU) [1]. Despite a well conducted medical treatment, worsening can occur in patients with acute exacerbations of COPD and lead to a decompensation phase. Acute respiratory failure can lead to the requirement of mechanical ventilation; in these cases, non-invasive ventilation (NIV) must be considered in order to avoid invasive mechanical ventilation and its related complications [2-4]. Studies by Plant *et al*. [4] offer very strong arguments for the delivery of NIV as soon as the patient develops an increase in PaCO_2 _and respiratory acidosis. Numerous patients are hospitalized with mild respiratory acidosis. Airway inflammation, bronchospasm and the increase in sputum volume are constant in these patients and are responsible for an increase in airway resistance and air trapping [5]. This air trapping and increased airway resistance result in hyperinflation and intrinsic positive expiratory pressure (PEEPi), which are common features during acute exacerbations of COPD patients and are responsible for increasing the work required to breathe and respiratory muscle failure. Methods of treatment directed against the onset of decompensation are attractive in theory, although the benefits of mucus clearance therapies have been regularly challenged [6-10]. Nevertheless, in COPD patients, there is a pathophysiological rationale for the use of a mucus clearance therapy. Hypersecretion of mucus, changes in mucus viscoelasticity and surface adhesion, and impaired ciliary function lead to entrapped mucus. Retained airway secretions can form mucus plugs and bronchial casts that cannot be expelled by coughing. Airway plugging causes impaired ventilation, resulting in lower ventilation-to-perfusion ratios. Increased airway resistance to airflow and air trapping result in hyperinflation of the chest and inspiratory loading of the respiratory muscles, leading to fatigue [11-14]. Two studies have shown that chest physiotherapy based on a mucus clearance strategy could represent a useful therapeutic option in exacerbations of patients with COPD [15,16].

Intrapulmonary percussive ventilation (IPV), intended for the therapeutic mobilization of bronchial secretions, has been primarily used in patients with cystic fibrosis in a stable state [17-22]. In addition, small pilot studies have shown the IPV device to be useful for increasing sputum production in patients with COPD [23,24]. IPV could offer a treatment directed against the onset of decompensation, specifically the increase in airway mucus that is responsible for increasing airway resistance.

We hypothesized that the use of IPV could be effective in avoiding further deterioration in patients admitted with acute exacerbations of COPD. In this prospective, randomized, controlled study, we compared the efficacy of standard medical treatment with supplemental oxygen and no ventilatory support to standard medical treatment with supplemental oxygen plus IPV in patients with acute exacerbations of COPD.

## Materials and methods

### Patients

Adult patients hospitalized in our ICU due to an acute exacerbation of COPD were prospectively studied. The experimental protocol was approved by the institutional review board of the hospital, and all patients or their next of kin provided written informed consent.

#### Inclusion and exclusion criteria

Patients were eligible for the study if they were admitted as an emergency with an exacerbation of COPD (on the basis of the clinical history, physical examination, and chest radiograph) [25], and a respiratory rate (RR) ≥ 25/min, PaCO_2 _> 45 Torr after the patient had been breathing room air for at least 10 minutes, and 7.35 ≤ pH ≤ 7.38 without metabolic acidosis. Exclusion criteria were: the requirement for emergency intubation for cardiopulmonary resuscitation, respiratory arrest, or in the case of rapid deterioration in neurological status (Glasgow coma scale [26] of ≤ 8); hemodynamic instability defined as a systolic blood pressure of less than 80 mmHg or evidence on electrocardiography of ischemia or clinically significant ventricular arrhythmias; failure of more than two additional organs; or tracheotomy, pneumothorax, facial deformity, or a recent history of oral, oesophageal or gastric surgery. Patients were randomly assigned to receive standard treatment or standard treatment plus IPV through a face mask. Random assignments were made with sealed envelopes.

#### Monitoring

Arterial oxygen saturation was monitored continuously with a bedside pulse oximeter (Oxisensor, Nellcor, Hayward, CA, USA); heart rate and RR were displayed on the screen of the monitor.

### Standard treatment

Patients assigned to standard treatment received oxygen with nasal cannulae to maintain a target oxygen saturation (recorded by pulse oximetry) of 88% to 92%. In all patients, the heart rate and RR were monitored continuously. The head of the bed was kept elevated at a 45-degree angle. The standard drug protocol consisted of nebulised salbutamol (5 mg every 4 h) or terbutaline, nebulised ipratropium bromide (500 μg every 6 h), subcutaneous heparin, corticosteroids (methyl prednisolone 2 mg/kg of body weight intravenously per day for three days; then decreasing doses of oral methyl prednisolone for 15 days), and an antibiotic [27]. Medication included the correction of electrolyte abnormalities.

### Intrapulmonary percussive ventilation

**Figure 1:**
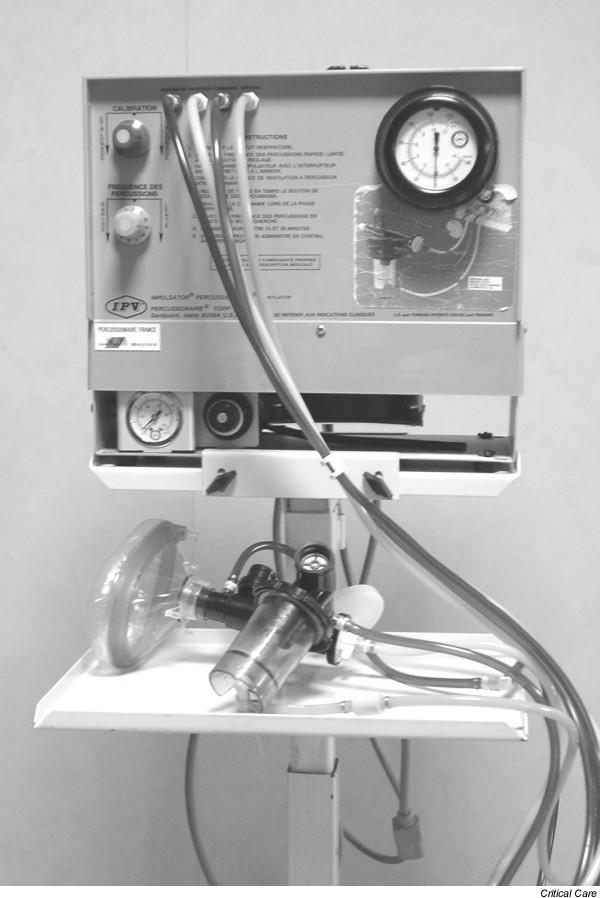
The intrapulmonary percussive ventilation (IPV) device (Percussionaire Corp., Sandpoint, ID, USA) and the full face mask used in the study.

**Figure 2:**
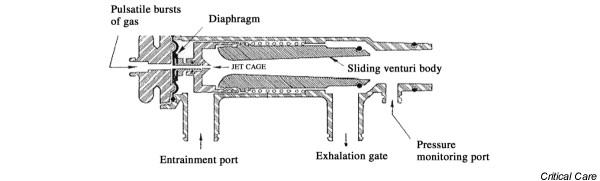
Schematic of the phasitron. The sliding venturi body moves back and forth to open and close the phasitron exhalation gate by an orificed diaphragm. Burst of air and aerosol are delivered to the patient during forward movement of the phasitron.

The IPV device was developed by Forrest M. Bird in 1979 (Fig. 1). IPV is a ventilatory technique that delivers small bursts of high flow respiratory gas into the lung at high rates. This causes airway pressures to oscillate between 5 and 35 cmH_2_O and the airway walls vibrate in synchrony with these oscillations. A unique sliding venturi called a phasitron (Fig. 2) is powered by compressed gas at 25 to 40 pounds per square inch and generates these oscillations in the range of 80 to 650 cycles per minute [28]. During inspiration the high frequency gas pulse expands the lungs and vibrates and enlarges the airways. This technique may be associated with nebulization [29] and has the potential to improve secretion clearance [30]. During the percussive bursts of air into the lungs, a continued pressure is maintained, while a high velocity percussive inflow opens airways and enhances intra-bronchial secretion mobilization.

Patients assigned to the IPV group received the same medication as the patients in the standard-treatment group with the addition of two sessions of IPV per day. No patient in either group received externally applied treatments designed to clear mucus. IPV sessions were performed by the specialized and trained respiratory therapist and delivered to the patient through a full face mask (La Cigogne, Pessac, France). The mask was adjusted and connected to the intrapulmonary percussive ventilator (IPV1 device, Percussionaire Corp., Sandpoint, ID, USA). After the mask had been secured, the percussions were delivered into the lungs of the patient. The frequency of the percussion was initially set at 250/minute and the peak pressure was initially set at 20 cmH_2_O. Frequency and peak pressure were adjusted for each patient to improve comfort and to be certain that the entire thorax was being percussed; this was done on the basis of visualization of external thoracic movements and perception of thrill on the patient's thorax. The inspiration-to-expiration ratio was adjusted to 1/2.5. During IPV sessions, the nebulizer delivered only NaCl 0.9%. Oxygen was fed into the mask to maintain oxygen saturation between 88% and 92%. The duration of each IPV session was 30 minutes. Between periods of IPV, patients breathed oxygen spontaneously while arterial oxygen saturation was continuously monitored. IPV sessions were stopped when the patients reached a RR of < 25/min and a pH > 7.38 in spontaneous breathing without worsening for 24 h.

### Success of therapy

Therapy was considered to be successful when it enabled the avoidance of both a worsening of the exacerbation and a decrease in pH to under 7.35 (which would have required NIV), and allowed the patient to be discharged from the ICU.

### Criteria for non-invasive ventilation

NIV was used as previously described [31,32] when patients were tachypnoeic with a RR of more than 25/min and a respiratory acidosis defined by a PaCO_2 _> 45 Torr and a pH lower than 7.35 without metabolic acidosis, at any time during the study.

### Follow-up

The RR and arterial-blood gas levels were recorded at base line and at the end of the first IPV session. On subsequent days, these data were obtained once daily during the morning. IPV session comfort was assessed using a five-point verbal-rating scale (comfortable, mildly uncomfortable, moderately uncomfortable, very uncomfortable, and intolerable). To assess the patient's severity of illness on ICU admission Simplified Acute Physiologic Score (SAPS II) was recorded for each patient [33]. Pulmonary function data were obtained from previous spirometric tests in 13 patients in the IPV group and in 14 patients in the control group (the last test was retained). For the other patients (three in the IPV group and three in the control group), reliable pulmonary function data were obtained within two months of inclusion in the study.

### Hospital stay

Patients were discharged from the ICU when their pH was higher than 7.38. Patients were discharged from the hospital when their clinical status and gas exchange were comparable to the stable state.

### Statistical analysis

The primary outcome variable was the avoidance of a worsening of the acute exacerbation leading to decompensation, defined by a pH < 7.35, and so the need for NIV at any time during the study. The secondary end point was the length of the hospital stay. Results are given as means ± standard deviation (SD). The group means were compared with the t-test. Repeated-measures analysis of variance was used to compare the partial pressure of arterial oxygen, PaCO_2_, RR, and bicarbonate values measured at base line and at the end of the first IPV session. A p-value of less than 0.05 was considered to indicate statistical significance. Analyses were done using SPSS statistical software, version 10 (SPSS, Chicago, IL, USA).

## Results

### Patient characteristics

A total of 81 patients who underwent episodes of acute exacerbation of COPD were admitted to our ICU over one 18 month period. Of these, 48 patients were excluded from the study: 40 patients required NIV because of a pH < 7.35 despite a well conducted medical treatment; and eight patients required immediate endotracheal intubation. In the end, 33 patients were included, of which 17 were randomly assigned to standard treatment, and 16 to IPV. The two groups had similar characteristics on admission (Table 1). The patients in both groups had respiratory disease of a similar severity; the functional steady-state characteristics were thus similar in the two groups. The same was true for the severity of exacerbation; there were also no significant differences in the SAPS II and the arterial blood gas levels between the standard treatment group and the IPV group. Chest X-ray findings were similar between the two groups. Four patients in the standard group and three patients in the IPV group had been previously non-invasively ventilated for a similar episode. The same medication was administered to the patients of both groups.

### Clinical outcome

As shown in Table 2, 6 out of 17 patients (35.3%) in the control group progressed to the point of requiring NIV, compared with 0 out of 16 in the IPV group (p < 0·05). The mean interval between entry into the study and decompensation was 48 ± 12 h for the six patients of the control group. NIV was successful every time and none of these six patients needed invasive mechanical ventilation. No patient included in the study died.

### Physiological outcomes

The values for the physiological variables in the IPV group on inclusion and at the end of the first IPV session are given in Table 3. IPV led to an improvement in PaO_2_, PaCO_2 _and RR (p < 0.05). No statistical difference was observed concerning the pH.

### IPV group

Patients assigned to the IPV group were given this method of treatment for a mean duration of 3 ± 1 days. The duration of IPV was half an hour twice daily. The mean frequency of the percussions was 250 ± 50 per minute. The mean peak pressure was 20 ± 5 cmH_2_O. IPV sessions were well tolerated. The median comfort score of IPV sessions was 2 (mildly uncomfortable). As judged by the physiotherapist in charge of the patient, mucus clearance was greatly improved by the application of the IPV.

### Hospital stay

The hospital stay was significantly longer in the group receiving standard treatment than in the group receiving IPV (7.9 ± 1.3 versus 6.8 ± 1 days, p < 0.05).

## Discussion

In this randomized trial, the use of IPV helped avoid further deterioration in patients admitted with acute exacerbation of COPD and mild acidosis. When compared with the patients who received standard treatment, the patients who received IPV had a lower incidence of NIV use and a shorter duration of hospital stay.

In acute exacerbations of COPD patients, NIV has profoundly changed the management and outcome of these patients [2]. The results of several prospective randomized controlled studies on NIV favor an early use of ventilatory methods as soon as the patient develops an increase in PaCO_2 _and respiratory acidosis [2,4]. To our knowledge, however, the potential benefits of NIV in COPD patients with mild respiratory acidosis have not been studied. We hypothesized that the use of IPV could be effective in avoiding further deterioration in this situation.

To date, few studies have been published on the use of IPV in adult patients with pulmonary disease. IPV has been used primarily, however, for the treatment of atelectasis and retained secretions in patients in a stable state, as occurs in a wide variety of conditions, including cystic fibrosis and neuromuscular disease [17-22]. Ravez *et al*. [23] studied the use of IPV in a small group of adults with chronic bronchitis. They found that total lung clearance of radioaerosol was enhanced with IPV therapy, but it was unclear how much IPV stimulated cough contributed to the observed benefit [23]. In addition, small pilot studies with the IPV device have shown it to be useful for the relief of lobar atelectasis and for increased sputum production in patients with COPD [24].

In this study, the most important question is: how does IPV improve the clinical status of patients with COPD?

### IPV is a mucus clearance device

Inflammatory cells are abundant in the sputum of patients with chronic mucus retention. These cells are able to release mediators that can alter the secretion and clearance of mucus. The end result is airway plugging, which causes bronchial obstruction resulting in atelectasis, impaired lung mechanics and gas exchange. There is a physiopathological rationale for the use of mucus clearance therapies because even small decreases in airway resistance may be important to achieve recompensation [11-14]. The benefits of mucus clearance strategies using physical and respiratory therapies, however, have been regularly challenged [27]. Three randomized, controlled trials of chest physiotherapy [6-8] and one observational study [9], showed that mechanical percussion of the chest as applied by physical or respiratory therapists was ineffective and perhaps even detrimental in the treatment of patients with acute exacerbations of COPD. None of these randomized trials reported any improvement in ventilatory function with respect to either forced expiratory volume in one second (FEV1) or functional vital capacity [6-8]. Furthermore, one trial described a significantly lower FEV1 in patients who received chest percussion therapy compared with controls [7]. No other adverse effects were reported. Does the lack of evidence, however, mean that there is a lack of benefit? Two studies have shown that chest physiotherapy based on a mucus clearance strategy could represent a useful therapeutic option in patients with exacerbations of COPD [15,16]. Bellone *et al*. [15] demonstrated that chest physiotherapy using a positive expiratory pressure mask in patients with mild acidosis (mean pH = 7.33) requiring NIV with pressure support could produce benefits in sputum clearance and could reduce the amount of time that the patient requires NIV [15]. Wolkove *et al*. [16] reported significant improvement in lung function after inhaled bronchodilator therapy, and the prior use of a mucus clearance device, compared to a sham mucus clearance device, improved the subsequent bronchodilator response in patients with stable COPD [16]. The mucus clearance device used (flutter device), promotes the clearance of sputum through the generation of low frequency pressure waves [16]. Both the IPV and the flutter device appeared equally effective in removing obstructing secretions from airways [34,35]. In our study, mucus clearance obtained by the application of IPV was judged greatly improved by the physiotherapist in charge of the patient; however, we did not measure the quantity of expectoration in both groups. Thus we can not conclude that IPV in addition to standard treatment increased the elimination of mucus to a significant increment.

### IPV theoretically increases mean airway pressure

PEEPi has been identified in patients with exacerbations of COPD because of severe airway obstruction [36-40]. In order to initiate inspiratory airflow, the respiratory muscles must generate a negative pressure equal in magnitude to PEEPi. The presence of PEEPi also implies dynamic hyperinflation, with consequent worsening of thoracic wall geometry and muscle length-tension relationships. This further increases the workload of muscles as their efficiency and mechanical advantage are reduced. The application of positive end-expiratory pressure (PEEP) at the airway opening should decrease the pressure gradient between the mouth and alveoli at the end of expiration and so reduce the inspiratory threshold load [38-40]. During the percussive sessions, IPV maintains an intrapulmonary pressure, which serves to stabilize airway patency. Improvement may occur via the beneficial effects of this intrapulmonary pressure, including the reduction of PEEPi and the amount of work required to breathe, which may allow respiratory muscles to regain efficiency.

### High frequency oscillatory ventilation like effects

Considering the effect of high frequency oscillatory (HFO) ventilation on gas exchange and breathing pattern, one can hypothesize similar effects with IPV. Indeed, any high frequency ventilation is a positive pressure ventilation, which would increase the airway pressure (Paw), induce a 'PEEP effect' and thus improve oxygenation [41]. Two mechanisms explain gas transport with respect to the clearance of CO_2 _during HFO,: convection and molecular diffusion. HFO maximizes CO_2 _removal primarily through facilitated diffusion [41]. The theoretical increase of mean Paw observed with IPV, however, is less important than the increase of Paw observed with HFO. Similarly, the frequency in HFO, generally set below 5 Hz, is more important than in IPV.

### IPV and lung volume

Any high frequency ventilation induces a PEEP effect that can increase lung volume. But according to the 'waterfall theory', if PEEPi is the result of expiratory flow limitation, application of extrinsic PEEP should decrease the pressure gradient between the mouth and alveoli at the end of expiration. This should be achieved without further hyperinflation. Several studies in patients during acute exacerbations of COPD have demonstrated this effect [36,38,39]. O'Donoghue *et al*. [40], however, found that only high levels of continuous positive airway pressure (CPAP) reduce PEEPi and indices of muscle effort in patients with severe but stable COPD, but only at the expense of a substantial increase in lung volume.

IPV sessions were well tolerated by the patients. Except for one episode of transient haemoptysis reported in a patient with cystic fibrosis [18], no serious adverse effects of IPV have been reported in previous studies [17-19]. All types of interfaces could be used to perform IPV sessions. On the basis of our previous experience with NIV in patients with acute exacerbations of COPD [31,32], we used a full face mask to perform IPV. This interface was well tolerated as most patients found the interface comfortable or only mildly uncomfortable.

The hospital stay was significantly shorter in the group receiving IPV than in the group receiving standard treatment. This result suggests that IPV may be a cost-saving measure. In addition, IPV sessions could be performed in general respiratory wards in COPD patients with mild respiratory acidosis and so could minimize the transfer of these patients to ICU. Further studies are needed to test this hypothesis.

Our study has several limitations. It is impossible to eliminate bias when a study cannot be blinded, so we have to be very careful concerning the shorter duration of the hospital stay in the IPV group. The study included only selected COPD patients with acute exacerbations who were treated in a single ICU. In the study, the control group didn't receive normal saline by nebulizer. Hypertonic saline has the potential of a mucotropic agent by shielding the excess of fixed negative charges that develop on mucins in airway disease [42]. Despite this, pharmacological mucus clearance strategies have not been demonstrated to shorten the course of treatment for patients with acute exacerbations of COPD, although there is a possibility that these agents improve symptoms [27]. Miro *et al*. [43], in a descriptive study, showed the benefit of CPAP sessions in seven COPD patients with acute hypercapnic respiratory failure in an attempt to avoid endothacheal intubation and mechanical ventilation. Goldberg *et al*. [44] conclude that the non-invasive application of CPAP to spontaneously breathing patients with severe COPD in acute respiratory failure decreases inspiratory effort and dyspnea while improving breathing pattern. End-expiratory lung volume remained stable at the lower levels of CPAP, with only modest increases at the higher levels [44]. These patients were at a more severe stage of decompensation. To our knowledge, there are no data concerning the utility of CPAP in acute exacerbations of COPD with mild respiratory acidosis. In a recent study, however, O'Donoghue *et al*. [40] found that only high levels of CPAP reduce PEEPi and indices of muscle effort in patients with severe but stable COPD, but only at the expense of a substantial increase in lung volume. It would be interesting, therefore, to perform another study with a control group of patients using CPAP to correct for lung volume change during intervention.

We have not evaluated the long term effect of IPV. Treatment with IPV was stopped when patients reached a RR of < 25/min and pH > 7.38 in spontaneous breathing without worsening for 24 h. No COPD patient worsened after the withdrawal of IPV. Also, we hadn't planned to evaluate IPV in patients recovering from their acute exacerbation. Another limitation of the trial was the small number of patients included. Further studies are also needed to determine the mechanisms of improvement with IPV in patients with acute exacerbations of COPD.

## Conclusion

This randomized controlled study shows that chest physiotherapy by IPV may prevent the deterioration of acute exacerbations of COPD with mild respiratory acidosis. The method we employed was well tolerated. In addition, the technique performed at an early stage could be a cost-saving measure. Further studies are needed to determine the long term effects of IPV and to clarify the impact of IPV in the management of patients with acute exacerbations of COPD.

## Key messages

• 	IPV led to a significant decrease in respiratory rate, an increase in PaO_2 _and a decrease in PaCO_2_.

• 	Clinical deterioration occurred in 6 out of 17 patients in the control group versus 0 out of 16 in the IPV group.

• 	The hospital stay was significantly shorter in the IPV group than in the control group.

• 	IPV is a safe technique and may prevent deterioration in cases of acute exacerbations of COPD with mild respiratory acidosis.

## Abbreviations

COPD = chronic obstructive pulmonary disease; CPAP = continuous positive airway pressure; FEV1 = forced expiratory volume in one second; HFO = high frequency oscillatory ventilation; ICU = intensive care unit; IPV = intrapulmonary percussive ventilation; NIV = non-invasive ventilation; PEEPi = intrinsic positive expiratory pressure; PEEP = positive end-expiratory pressure; RR = respiratory rate; SAPS II = simplified acute physiologic score; SD = standard deviation.

## Competing interests

The authors declare that they have no competing interests.

## Authors' contributions

FV, NHB, AB, GGB, HG, DG, GH conceived of the study, and participated in its design and coordination and helped to draft the manuscript. LRS participated in the design of the study and performed the statistical analysis.

## Figures and Tables

**Table 1:** Characteristics of COPD patients assigned to receive intrapulmonary percussive ventilation or standard treatment at inclusion

	IPV (n = 16)	Standard treatment (n = 17)	p-value
Age (years)	69.2 ± 6.0	70.2 ± 5.0	NS
SAPS II	25.4 ± 6.0	25.4 ± 4.0	NS
Hb (g/dl)	13.7 ± 1.3	14.0 ± 1.4	NS
HR (beats/min)	117 ± 16	115 ± 19	NS
RR (breaths/min)	36 ± 2	36 ± 3	NS
SBP (mmHg)	141 ± 15	143 ± 16	NS
pH	7.37 ± 0.01	7.37 ± 0.01	NS
PaCO_2 _(Torr)	57.6 ± 4.5	58.0 ± 3.0	NS
PaO_2 _(Torr)^a^	56.9 ± 3.0	56.7 ± 3.0	NS
Bicarbonate (mmol/l)	33 ± 3	33 ± 3	NS
FEV_1 _(%)^b^	39 ± 7	38 ± 8	NS

^a^Patients received oxygen with nasal cannulae to maintain a target oxygen saturation (recorded by pulse oximetry) of 88% to 92%. Mean oxygen flow was 2.0 ± 0.5 l/min and 2.0 ± 0.5 l/min in the IPV group and the control group, respectively (p = NS).

^b^FEV1 was obtained from previous spirometric tests in 13 patients in the IPV group and in 14 patients in the control group (the last test was retained). For the other patients (three in the IPV group and three in the control group), reliable pulmonary function data were obtained within two months of inclusion. FEV_1_, forced expiratory volume in one second; Hb, hemoglobin; HR, heart rate; IPV, intrapulmonary percussive ventilation; RR, respiratory rate; SAPS II, simplified acute physiologic score; SBP, systolic arterial blood pressure; NS, not statistically significant.

**Table 2:** Clinical outcome of COPD patients assigned to receive intrapulmonary percussive ventilation or standard treatment

	IPV (n = 16)	Standard treatment (n = 17)	p-value
Worsening of exacerbation with pH < 7.35 (NIV required) (%)	0 (0)	6 (35.3)	<0.05
Hospital stay (days)	6.8 ± 1.0	7.9 ± 1.3	<0.05
Hospital death	0	0	-

**Table 3:** Values of arterial blood gas and respiratory rate

	IPV (n = 16)		
	Inclusion	Post IPV session	p-value
Arterial pH	7.37	7.38	NS
PaCO_2 _(Torr)	57.6 ± 4.5	53.5 ± 2.3	<0.05
PaO_2 _(Torr)^a^	56.9 ± 3.0	61.0 ± 0.8	<0.05
RR (breaths/min)	36 ± 2	31 ± 2	<0.05

Measurements were taken at inclusion and at the end of the first intrapulmonary percussive ventilation session (post IPV session; in the thirtieth minute). ^a^Oxygen flow was not changed between both periods and was 2.0 ± 0.5 l/min. IPV, intrapulmonary percussive ventilation; RR, respiratory rate; NS, not statistically significant.
